# ‘All in good faith?’ An ethno-historical analysis of local faith actors’ involvement in the delivery of mental health interventions in northern Uganda

**DOI:** 10.1177/13634615221149349

**Published:** 2023-02-06

**Authors:** Elizabeth Storer, Costanza Torre

**Affiliations:** 1 Firoz Lalji Institute for Africa, 4905 London School of Economics and Political Science, UK

**Keywords:** mental health, local faith actors, northern Uganda, refugees, humanitarianism

## Abstract

Faith actors have become increasingly significant in the field of global mental health, through their inclusion in the delivery of psychosocial support in humanitarian settings. This inclusion remains empirically underexplored. We explore historical and contemporary activities of local faith actors in responding to mental disquiet in northern Uganda. Given pre-existing roles, we question what it means when humanitarians draw on faith actors to deliver mental health and psychosocial support (MHPSS) in conflict-affected settings. We argue for a recognition of faith actors as agents operating within a therapeutic marketplace, which on occasion links suffering to social inequality and exclusion. We show, moreover, that the formal inclusion of Christian actors within MHPSS may not equate to the enforcement of rights-based values at the core of international ideas of protection.

## Introduction

Since the 1990s, the field of humanitarian protection has undergone profound shifts. On the one hand, mental health and psychosocial support (MHPSS) interventions aimed at improving the psychological wellbeing of crisis-affected populations, have come to constitute an established branch of humanitarian assistance (Jones & Ventevogel, 2021; Tol et al., 2020). On the other hand, humanitarian policy and discourse has increasingly advocated for a move towards the localisation of the operational delivery of assistance. By virtue of the legitimacy which they often retain among their communities, local faith actors (LFAs) have become particularly significant in mediating the distribution of humanitarian assistance (Thomson, 2014; Wilkinson et al., 2022). At a time at which MHPSS interventions are burgeoning thanks to the influence of the field of global mental health, humanitarians have begun to involve LFAs in the provision of MHPSS.

Critics have pointed out that international aid often instrumentalises LFAs, imposing requirements to fit within international programmes (Ager & Ager, 2011; Jones & Petersen, 2013). As such, extensive policy and scholarship has focused on how to localise assistance (Wilkinson et al., 2019; OECD, 2017). The potential implications of faith-based alliances have been left out of humanitarians’ calculations. We suggest it is important to critically consider the complex (and sometimes problematic) social processes through which LFAs become legitimate. LFAs’ involvement in dealing with forms of mental suffering is far from novel, and although faith can benefit individuals’ psychological wellbeing, LFAs’ actions also have an important role in governing the social and moral landscapes in which they are situated, sometimes through normative and exclusionary practices.

Although a rich anthropological literature offers in-depth analyses of the socio-moral work of religious actors, this scholarship is frequently disregarded by advocates for the inclusion of LFAs in MHPSS (Ager et al., 2018). Furthermore, bound by Euro-American scripts of mental health and symptomatology of illness, humanitarians and clinicians assume accounts of possession and witchcraft to be distant from their work. However, there is a distinct overlap in such experiences for people who are the target of humanitarian aid (Whyte, 1997). By uncritically positioning LFAs as ‘brokers’ for the delivery of assistance, current humanitarian policies risk overlooking, and indeed facilitating, forms of violence and oppression that are well-documented in anthropological scholarship. Such a risk, which directly contradicts humanitarian mandates, makes these new alliances with humanitarian actors worthy of critical analysis.

In this article, we offer a critical analysis of the inclusion of LFAs in the delivery of mental health assistance, rooted in ethnography and historical practice. We do so not through assuming the primacy of humanitarian principles, but through examining processes on the ground. As such, we reverse the donor–recipient gaze, centring ethnographic evidence and departing from an appreciation of what role(s) LFAs already play in the field of mental health, thereby extending our reflection to what this means for humanitarian actors pushing for closer partnership with them. Specifically, we depart from extended engagement with the pre-existing everyday work of religious actors in addressing mental disquiet across conflict-affected populations across northern Uganda, a region where we have worked continuously between 2015 and 2021. Acknowledging the diversity of actors connected to faith, we follow Wilkinson's categorisation of LFAs as ‘groups of individuals, and individual religious leaders with different levels of power and religious affiliation across local and national levels’ (2018, p. 113). Although several faiths are professed in northern Uganda, Christian actors retain by far the most widespread presence, with Muslim ones representing an historically small minority (Alava, 2017; Ward, 2001). Therefore, in this article, we focus particularly on the actions and histories of Christian faith actors.

This article begins by analysing the shifts in global humanitarian policy that push for the inclusion of LFAs in the delivery of mental health interventions. To situate LFAs within the different actors involved in healing psychological suffering in northern Uganda, we then outline the therapeutic landscape of the region, and introduce our research sites – Lamwo District, Acholiland and Arua District, West Nile. In these diverse but deeply connected spaces we explore how the actions of LFAs, involved in redrawing moral–social boundaries through cleansing possession and witchcraft, have had widespread social consequences for recipient groups. We conclude with a discussion of the implication of overlooking these activities for humanitarians.

## Including LFAs in the delivery of mental health

Despite roots in a long history of Christian missionary efforts, and despite fundamental links to altruism and compassion, the humanitarian system is generally understood in secular terms (Barnett, 2011; Ferris, 2011). Over the past decade, however, the role of faith in humanitarian work has become the object of renewed interest, in what Wilkinson et al. (2022) call a ‘turn to religion’ among international aid actors. Scholars have argued for the recognition of religion and spirituality as central to the lives of crisis-affected groups (Ager & Ager, 2011; Wurtz & Wilkinson, 2020).

The rediscovery of the role of faith has coincided with the ‘localisation’ of aid (UNHCR, 2020; WHO, 2020; World Vision, 2020). LFAs, it is argued, are trusted and knowledgeable about their communities, positioning them as ideal humanitarian ‘brokers’ to deliver humanitarian aid (Wilkinson et al., 2019). Several ‘toolkits’ and guidelines have been produced to achieve such collaborations (French et al., 2018; Watson et al., 2020), while the United Nations High Commissioner for Refugees (UNHCR) has described LFAs’ inclusion in humanitarian activities as a ‘journey of mutual discovery’ (UNHCR, 2013, p. 6). Although the consensus is to regard both development and religion as inherently good things (Rakodi, 2007), the existing literature tends to portray faith actors as a homogenous group, disregarding their local histories (Ferris, 2011). In addition, although much development literature invokes normative assumptions that faith actors can easily be harnessed to deliver projects, evidence of effectiveness is lacking (Olivier et al., 2015; Winiger & Peng-Keller, 2021).

Perhaps unsurprisingly, owing to the established links between psychosocial wellbeing and spirituality (Hatala & Roger, 2021; Schafer, 2010), one of the most visible entanglements of faith and aid delivery has been found in the delivery of mental health assistance. The past two decades have seen the growth of the academic and policy field of global mental health, which has emphatically advocated for an increase of efforts in delivery of psychological assistance in the Global South (Tol et al., 2020), and which has contributed to establishing clear links between mental health and development through the inclusion of the former in the UN Sustainable Development Goals (Bemme & Kirmayer, 2020; Mills, 2018; White et al., 2017). As such, MHPSS has been established as a mainstream component of humanitarian aid worldwide (Jones & Ventevogel, 2021).

MHPSS practitioners have long advocated for the formal inclusion of religious actors in the delivery of psychological assistance (e.g., IASC, 2008).^1^ A growing clinical and academic literature argues for the need of faith-sensitive MHPSS and for establishing collaborations with LFAs (Ager et al., 2018; Harsch et al., 2021; Rutledge et al., 2021; Trotta & Wilkinson, 2019). Faith-based organisations, including the Lutheran World Federation, World Vision and Islamic Relief, have increasingly started offering mental health services and lay mental health techniques such as psychological first aid and psychological counselling trainings to religious leaders (Featherstone, 2015; Türk et al., 2014; World Vision, 2020). In northern Uganda, several mental health programmes are already delivered by faith leaders (Ager et al., 2014).

A large body of anthropological literature has examined in-depth the work of religious actors in social healing (i.e., Behrend, 2011; Mogensen, 2002; Vokes, 2013). This literature tends to be side-lined by humanitarian actors, yet attentive consideration of the anthropological evidence detailing faith actors’ deep historical engagement with local social fabrics provides an extensive repertoire to think through their involvement in humanitarian mental health provision. Based on our ethnographic work in northern Uganda, we seek to analyse and extend this evidence base focusing on the pre-existing work of LFAs.

We ask: What does it mean to build collaborations with LFAs who have pre-existing social roles and relationships in communities of interest to humanitarian workers? What are humanitarians legitimating through engaging with LFAs? To answer these questions, we depart not from preconceived ideas of how assistance should work, but from understanding pre-existing approaches to manage psychological suffering in conflict-affected spaces (Macdonald & Allen, 2015). In what follows, we consider therefore joint histories of trauma management and LFAs’ work in dealing with psychological distress in northern Uganda.

## Researching trauma healing in northern Uganda

A region with a deep history of conflict, as well as both internal and cross-border displacement, northern Uganda has long served as a testing ground for new forms of delivery of humanitarian and development assistance. Such interventions have frequently been abstracted from the social context in which they inevitably become entangled.

Recent upheavals have coincided with rising donor-interest in psychosocial suffering, and northern Uganda has become a case study par excellence for the management of post-conflict trauma. Multiple studies have analysed the after-effects of the Lord's Resistance Army (LRA) bush war (1986–2006), which was characterised by extensive violence, displacement and abduction (Dolan, 2009; Finnström, 2008). The aftermath of war was characterised by extreme psychosocial suffering, manifesting in high rates of trauma and depression (Annan et al., 2011; Roberts et al., 2008), alcoholism (Mehus et al., 2021; Meinert & Whyte, 2020), domestic and sexual violence (Baines & Rosenoff Gauvin, 2014; Porter, 2016), spiritual problems (Meinert & Whyte, 2017) and alarming rates of poverty (Owori, 2020). A widespread ‘trauma discourse’ (Argenti-Pillen, 2000) provided a moral and financial rationale for the implementation of countless humanitarian mental health interventions targeting pot-traumatic stress disorder and depression in Acholiland (Torre et al., 2019).

The types of therapy assumed to constitute post-conflict healing have been numerous and diverse. In part, this presentation of diversity spans from epistemological and evidentiary divides within academic disciplines. Csordas (2021) notes the disconnect between the evidence-based psychiatric approaches of global mental health and anthropological perspectives grounded in local vernaculars and experiences. Despite well-noted divergences, firmly rooted in Western psychiatric frameworks, interventions established a direct correspondence between forms of mental and spiritual affliction suffered in the region and psychiatric labels. A large cluster of studies focused on the translation of Acholi experiences into Western clinical terms (Harnisch & Pfeiffer, 2018; Neuner et al., 2012).

Yet, medical anthropologists have frequently questioned the transferability and ethics of exporting Western interventions into non-Euro-American contexts (Bemme & Kirmayer, 2020; Kienzler, 2008; Summerfield, 2012). Wary of the risks, healing has been studied with reference to local cosmological worlds, rather than to clinical diagnoses. Perhaps intentionally writing out the impacts of humanitarian presence, anthropological studies have tended to focus on the hybrid tapestry of local actors (elders, diviners and faith actors) who have attended to trauma through sociocultural processes to assuage distress. Owing in part to the metaphysical aspects of the war in Acholiland, including the LRA’s intentional strategy of forcing recruits to conduct violent, spiritually polluting acts, anthropological research has often focused on the management of hauntings known traditionally in Acholi as *cen*, a form of spiritual disturbance often understood to bring madness (*apoya*) (Meinert & Whyte, 2017; Victor & Porter, 2017; Williams, 2021).

Studies of this nature have made relevant contributions to the current research. First, ethnographies have shifted from an analysis of discrete interventions (in clinical or religious settings) to highlight the processes through which individuals heal. Managing trauma is revealed to be a social conversation, or a ‘therapy pathway’, in which people follow the cause of disquiet and seek remedy in homes and communities, as well as in treatment settings (Victor & Porter, 2017). Second, ethnographers have highlighted that therapy options are structured by market competitions: Hilhorst and Jansen (2010) have described these options as an arena, and Williams and Schulz (2021) have referred to northern Uganda as a ‘marketplace of post-conflict assistance’. Third, participation in this marketplace, particularly when flows of finance become involved, changes the legitimacy of therapy and actors (Komujuni & Büscher, 2020). Indeed, Allen reminds us that the internal dynamics of healing competition predate colonial rule (1991).

These studies include LFAs as one actor among many. Despite a few notable exceptions (e.g., Alava, 2022), studies on faith tend to emphasise the individuated aspects of interventions that resemble Western therapies (Williams & Meinert, 2020). Constrained by time, most studies have focused on a single church or religious group, and few inquiries have examined individual therapeutic outcomes in conversation with their wider social, moral and political actions. Inspired by the approach that recognises contestations within healing arenas, we reflect on LFAs’ pre-existing social dynamics, and the unintended consequences of their reinforcement through humanitarian alliances.

### Our connected research sites

We draw on ethnographic data collected through interviews, focus groups, observations and informal conversations between 2016 and 2018 in Arua District, West Nile sub-region, and between 2018 and 2020 in Palabek refugee settlement, Lamwo District, Acholiland.

These sites provide fruitful contexts within which to examine the implications of humanitarians’ recent alliances with LFAs. In Lamwo, we explore alliances that form out of the involvement of LFAs in delivering MHPSS in Palabek refugee settlement, currently home to over 68,000 predominantly Acholi-speaking refugees from South Sudan. In Arua, we draw on the everyday work of LFAs in attending to mental disturbances in Lugbara-speaking communities living near the border of the Democratic Republic of Congo. These groups have been of interest to scholars of humanitarianism for several decades, precisely because of the absence of humanitarian intervention and the improvisation of local solutions (Crisp, 1986).

#### Humanitarian interventions: An expanding therapeutic marketplace?

To the mental health professional, treatment options in Arua and Palabek look very different. Despite being classified as a peaceable, ‘post-conflict’ space, in Arua Town and the wider sub-region mental health services have achieved relatively limited penetration; here, LFAs and ‘traditional’ actors dominate the healing marketplace. In this sense, West Nile resembles wider Uganda, which, with fewer than 1 psychiatrist per 100,000 people, is among the least-equipped countries in the world when it comes to mental healthcare (WHO, 2017). In 2018, a single psychiatrist worked at the psychiatric unit within Arua's main hospital, which serves the entire West Nile sub-region. Of those admitted, he estimated that 75% discharged themselves prior to completing treatment, instead ‘going home’ to consult herbalists and other religious specialists. Faith features highly in Aruans’ pragmatic management of mental affliction.

By contrast, Palabek refugee settlement, founded in 2017 to shelter people fleeing the resurgence of conflict in South Sudan, serves as a spatial expression of recent shifts in humanitarian policy (Torre, 2021). Various non-governmental organisations implement mental health and psychosocial interventions, and psychiatric nurses and officers operate daily within the primary clinics in the settlement.

Such attempts at establishing mental health caregivers as legitimate were often not enough to convince patients to rely on them: ‘When it comes to mental illness, we are people's last choice … they will try everything else before they come to us’, one mental health specialist explained. Refugees in Palabek seemed to seek the advice of mental health professionals only in rare cases, often only if explicitly referred to them by humanitarians. As in Arua, in Palabek refugees did what they had done before encountering humanitarian mental health interventions; they either turned to the church, which here too retains a huge presence in the management of various forms of mental distress, or they consulted traditional healers. Across both sites, faith-based interventions held more resonance than MHPSS.

#### Christian legacies and convergences in West Nile and Acholiland

Both Arua and Palabek share important Christian legacies. Populations were first exposed to Christianity through aggressive European evangelism spanning the late 19th to early 20th centuries (O’Byrne, 2017; Storer et al., 2017). Throughout the late colonial period, a faith associated with urban elites gradually spread over parts of the countryside, assisted by the arrival of the East African Revival from the late 1940s, which encouraged worship in African languages and styles (Lloyd, n.d.; Wild-Wood, 2010). Resistance remained, in part because of faith actors’ disavowal of ancestor veneration.

Post-independence in 1962, war catalysed rates of Christian conversion. During post-Amin reprisals in West Nile from 1979, the ongoing war in South Sudan and the LRA bush war in Acholiland, Christian leaders played significant roles in materially and spiritually protecting populations. The centrality of LFAs in providing war-time support, accounts for their legitimacy as arbiters of psychological after-effects. Repeating the activities of West Nilers’ exile in Congo, Christian clergy in Palabek now offer support to a new generation of refugees suffering displacement. Since the 1990s, Charismatic Catholic and Pentecostal churches have appeared across Uganda and South Sudan, spreading from towns to rural areas. Movements have been fuelled both by interdenominational competition and by an acceptance of healing gifts. These movements have further amplified competition in the marketplace for healing.

In this context, we thus find deep continuities between the Christianisation of scripts of mental affliction in South Sudan and northern Uganda. O’Byrne's (2017) ethnographic study of South Sudanese Acholi people in Pajok, prior to their displacement to Uganda in 2017, provides an extensive portrait of the work of independent churches in managing possession and other health crises. This study echoes the forms of spiritual cleansing documented by Storer in Arua and by Torre in Palabek. Although according to humanitarian classifications populations would occupy different stages of a conflict/post-conflict continuum, options for managing mental distress are markedly similar. We now turn to an exploration of contemporary and historical interventions.

### Social therapies: LFAs’ work in managing mental disquiet

Across our research sites, LFAs from both the Anglican and Catholic faiths were continually involved in the management of manifestations of mental disquiet. Rather than invoke Western vernaculars, in both Arua and Palabek mental suffering was often translated as a lack of peace, ‘overthinking’, sadness, ‘madness’, receiving revelations, hearing voices, body weakness, night terrors and visions, or possession-like episodes.

Across northern Uganda, mental health is rarely defined symptomatically, but is rather defined with reference to the social context surrounding individual cases. Discerning the root cause in a web of potentialities plays a fundamental part in the process of healing. Though the techniques within the process often involved a significant amount of improvisation, Lugbara and Acholi people suffering mental disquiet regularly sought counselling from LFAs who were either local pastors or more distant leaders reputed to have healing hands and prayers. Counselling sessions were often one-to-one, with some religious institutions offering designated times for weekly counselling. Most who sought counselling did so over periods of weeks and months. If after that time the problem was not deemed to be ‘solved’, alternative specialists were sought.

In cases of ongoing mental distress, collective attention or ‘home prayers’ were called. Here, either members of a sufferer's ‘home’ congregation or more itinerant charismatic factions were sought to pray for the afflicted. With the severity of the cases grew the importance of identifying the root cause of the distress, and in many cases, LFAs would sift through potential causes with the sufferer and their family members. In the Catholic tradition, this was sometimes done through a ‘Family Tree Healing’ technique, where families gathered to pray and ‘dig’ for the root cause of suffering (Storer, 2021). Important to note is that this process was not performed exclusively for – but often included – persistent cases of mental disquiet. After several self-led sessions, a Charismatic Catholic or Pentecostal priest led a group session aimed to reveal the cause of suffering.

Depending on the persuasion of the prayer group, these performances could become ecstatic, involve the laying-on of hands or feature visions that revealed the causes of suffering. In more extreme cases, where a sufferer's symptoms included possession, name calling, falling into a trance or where their activities potentially posed harm to families or wider communities, a more urgent intervention resembling what would be termed an ‘exorcism’ in the European vernacular, was sought. Often, the encounter was dramatic, involving the LFA ‘casting’ out demonic forces in the name of Jesus or the Holy Spirit. The sufferer would roll, cry and/or shout, indicative of the spiritual battle between evil and good spirits. Although exorcisms were often performed in people's homes, owing to recent demand a priest at a popular Catholic healing centre in Arua delivered hundreds of people each week.

At the individual level, it would be possible to find examples of patients who experienced relief from these sorts of interventions, although for many this required several sessions. It is possible to draw equivalent conclusions as other medical anthropologists who have found prayer and religious healing to be a cathartic process for relieving trauma (Frank & Frank, 1993; Luhrmann, 2012; Williams, 2021). One could, too, draw parallels between the therapeutic methods of psychiatrists, who take family histories to reveal genetic predispositions to forms of mental illness. As one Anglican reverend explained, in a way that closely resembled a clinical psychiatric narrative: ‘When people think they are possessed by a demon, they explain their symptoms – usually hallucinations, of course in the church the vision is a common thing … We treat them, evaluate them further, realise the cause, and control the hallucinations.’ Although processes can be individualised, the therapeutic process is often geared to revealing the social causes of distress. As is the case for many alternative healers, mimicking biomedical procedure and systematising diagnoses provides a means to legitimise spiritual interventions.

Returning, however, to the current debates in humanitarian circles, we contend that the impact of LFAs’ actions on mental distress goes far beyond an individual dimension. Indeed, the diagnoses of LFAs frequently tied symptoms to the social fabric, often ascribing mental disturbances to unbalanced social relations. For example, cases of possession were often linked to bewitching, brought on by tense social relations within the sufferer's social world. In doing so, LFAs reiterate longer histories, whereby their presence has transformed the management of mental distress. Colonial records report the increasing nature of possession and ‘madness’ throughout British rule (p’Bitek, 1964). During this time, fierce struggles over the management of such cases ensued, whereby the interventions of diviners were increasingly displaced by LFAs. Today, Christian actors have the monopoly on managing this type of affliction, and now compete not with traditional religions, but with each other in therapeutic marketplaces (Verginer & Juen, 2019).

Indeed, in Uganda and beyond, the histories of imposing Christianity are entangled with transformation both in the concepts and the management of mental health. Through the engagement of LFAs in managing possession and other local expressions of mental disquiet, mental conditions were pulled into the social diagnostic realm (Verginer & Juen, 2019). Rather than being linked to amoral spirits in the wild, illness increasingly became attributed to harm caused by family, clan or proximate neighbours, as well as by social practices that deviated from religious directives. Indeed, these histories of entanglement are present even in local languages. For example, in Lugbarati madness (*zizaru*) is derived from *ziza*, the term that now denotes Christian prayer. Because their action established direct links between causes of mental illness and Christian moral codes, therefore, LFAs must be understood as social healers, opening up the diagnosis of mental disquiet to social malevolence in ways that female diviners either cannot or would not, since this realm of blame often proves risky for individual therapists (Storer, 2021).

However, LFAs’ establishment of moral boundaries often involves perpetuating forms of violence and exclusion, which a narrow focus on individual impacts risks overlooking. In what follows, we present empirical findings highlighting two realms in which this is particularly prevalent, and encourage humanitarian workers to remain keenly aware of these aspects while relying on LFAs as brokers for localisation agendas.

#### Gendered divisions in therapy

As noted above, in northern Uganda manifestations of mental illness that LFAs have become involved in managing often included forms of spirit possession. However, this work has distinct effects across genders, which deserve to be kept in mind by humanitarian policy that seeks as well to diminish gender-based violence. Across northern Uganda, since possession manifests most commonly in women, and often in individuals suffering from social ostracisation, ethnographers have interpreted it as a manifestation of gendered expressions of power (Allen, 1991; Finnström, 2009; Middleton, 1960). The expression of possession has come to be understood as a reflection of the structural marginality that women face in deeply patriarchal contexts, where gendered norms have been vigorously reasserted following war (Baines & Rosenoff Gauvin, 2014).

Accordingly, in northern Uganda the reliance on Christian leaders to treat cases of possession is most prevalent among women and, latterly, other marginalised social groups (Storer, 2021). While in part, this reflects the actual incidents of possession, it also reflects sufferers’ financial options – those who consulted LFAs often lacked funds for transport and/or clinical therapy, or for purchasing an animal to appease elders and attend to illness through customary pathways. Moreover, patriarchal norms often dictate the uneven distribution of resources within homes, with women lacking access to funds for alternative care options. Increasingly, men leave women altogether – and with traditional marriage rites often undone, women cannot turn to their extended relatives for help (Baines & Rosenoff Gauvin, 2014).

Crucially, those women who visited LFAs often experienced possession symptoms alongside domestic violence or extreme poverty. Yet oftentimes, the explanations for suffering given by LFAs linked mental disquiet not to structural determinants of health, but to suffering due to a spirit, demon or witchcraft practised by a proximate associate. In all instances, mental disquiet was linked to sociocultural idioms of distress that focused on a sufferer's actions, or their close relations with others. For example, one married woman in Arua explained: ‘I entered the charismatic through the persecutions in my home. I was suffering from so many domestic problems. There were so many sicknesses in my home … When I entered this association, the lord gave me the gift through the holy spirit – that sickness stopped in the home.’

Things were similar in Palabek, where the case of Aber's possession illustrates the gendered asymmetries that can arise within LFAs’ interventions. In early 2019 Aber's husband abandoned her and her two children in the settlement, presumably to go back to South Sudan. Generally good-humoured and well-liked by her neighbours, she started drinking. During the day, she often wandered drunk through her block; rumours spread that men had been visiting Aber at night, which was understood by her neighbours as indicative of her moral dissolution. Soon afterwards, she started experiencing violent spirit possessions. In these cases, despite the fact that she was not a regular churchgoer, neighbours and members of the nearby Anglican church gathered to perform ‘home prayers’. This entailed sitting or standing around Aber, who was made to lie down outside on a mat, and emphatically praying with their hands hanging over or directly touching her body, shouting and ordering the devil to leave her.

The second time Aber became possessed, in a particularly violent episode witnessed by Torre in May 2019, she was transported to the local church where the prayer continued with the help of the pastor. When asked a couple of days later why she thought this was happening to her, still shaken up and suffering from muscle pain from the strain that the possession had put on her body, Aber said she was not sure. However, she added, she had been ‘overthinking’ – a common idiom for mental distress in Palabek – since her husband abandoned her. Because he had left her with nothing, she struggled to rely on the scarce humanitarian assistance alone.

The church members’ explanations were quite different, and focused on Aber's individual behaviour rather than on the external circumstances that she felt had deeply impact her life: ‘If you drink alcohol, the *cen* will not go away; the devil also likes alcohol’, one of her neighbours said. One pastor from the congregation that had been praying for her commented that she had brought her issues on herself: ‘You need to keep your door closed to the devil at all times. But if you drink, and have sex with different men, you are opening the door to it.’ Although previously she could often be found sitting and chatting at someone's home, after this second episode things seemed to change. Aber seldom left her compound, drinking frequently and growing more isolated as her neighbours also stopped visiting her.

The narrative upheld by the pastor that dealt with Aber's case placed the blame for the suffering she was experiencing entirely upon herself. Yet the stigma around her situation promoted by LFAs’ explanations resulted in her increased isolation, causing her mental health to deteriorate significantly. Often, faith actors invoke explanations that write out both past and present violence, to instead place the onus on the individual themselves or their associates (Tankink, 2007). Especially in normative and patriarchal societies, women are particularly affected by the consequences of these healing models, because the structural determinants of their psychological suffering are consistently erased from view in the work of LFAs. Rather than link violence or abandonment to mental affliction, within churches causes for suffering are often found in individuals’ own behaviours, and solutions in offering forgiveness and the reformation of social conduct. Aber's case – which was managed in the community, rather than through consulting mental health services – shows the extent to which the work of faith actors is embedded in, and indeed reinforces, wider structures of power that delegitimise the suffering of those more likely to be negatively affected by them. This case, embedded as it was with social norms and expectations, was managed in the same geographical arena where advocates of MHPSS – and often the same faith actors involved – were pushing for individuated norms associated with mental health interventions.

#### Excluding ‘others’ in healing marketplaces

The everyday work of social and moral boundary-making, whereby LFAs associate possession with others, is also layered with more complex forms of othering and exclusion. Although LFAs may be conversant in multiple vernaculars of mental health, often, individuals position themselves as social arbiters, dictating the right way to heal in therapeutic marketplaces though the exclusion of other therapists. To understand this, it is important to consider the therapeutic marketplace, and social conversations about faith and healing that present seeking care from LFAs as godly, and condemn other specialists as ‘witchdoctors’. This too is a term with a colonial history, introduced by missionaries to – at various junctures – condemn all non-Christian healers including prophets, diviners, elders, herbalists and latterly Pentecostal preachers. The vociferous nature of these campaigns to criminalise particular forms of healing cannot be understated – throughout the research period, the subject was continually referenced by faith, government and health authorities during sermons, over the radio, and in the local and national news. Although quests to dictate the way to heal may be well-meaning within faith-based ontologies, such exercises often hold potential for social exclusion.

To appreciate the nature of this exclusion, it is important to note that in northern Uganda, Christian conversion and allegiance has often been premised on denouncing ‘other’ religious practices. During Protectorate rule, European missionaries presented Christianity as an antithesis to ancestral ‘worship’. Although the veneration of ancestors was, in fact, connected to healing and the maintenance of social boundaries, fluid cultural practices were described as ‘pagan’ and ‘satanic’. In the early independence periods, educated Lugbara and Acholi converts, as well as Revivalists, were often involved in the destruction of ancestral shrines, resulting on occasion in violent resistance. Wars and exile – when elders where distanced from the shrines – effectively brought an end to the uncontested legitimacy of ancestors within concepts of healing. The significance of this shift cannot be overlooked: it is into the space that elders once occupied, dissolved through the gradual destruction of shrines, that Christian actors have defined themselves as moral authorities able to manage suffering. As is the case with possession, where Christian actors replaced the secret work of diviners, here too, LFAs have socialised lineage healing.

Yet war and displacement saw the abandonment of shrines. Since the 1980s, LFAs in Arua have found new ‘others’ against which to measure the faith of believers and from whose forms of ‘evil’ to offer protection. Initially, this reaction was against ‘witches’ who were often traders who had prospered from cross-border commerce during war, reportedly from acquiring ‘underwater’ power. One elderly lady who had returned to Arua from Congo explained, ‘[Though] there were now people prophesying against witchcraft and going under the water.’ Split between Christian logics of good and evil, the same marketplace that governs healing interventions is also believed to offer opportunities for harm.

Accordingly, new interventions have emerged to manage these threats. Eloquently described by Behrend (2011) in Torro (Eastern Uganda), the participation of Charismatic Catholics in cleansing new forms of witchcraft through prayers and exorcism during the past decade, constructed the very world it described through its attention to producing evidentiary claims. Across northern Uganda, in contexts of repeated physical and mental suffering for which witchcraft (or a witch) is deemed responsible, Christians decamp to a village to pray and cleanse the area. Increasingly, these groups were responding to outbreaks of possession among women and school children, and sometimes among young men (Victor, 2019). Prayers could last up to a week, and Christians often received revelations of the cause of affliction, or extracted evidence – herbs, hair, bottles – to prove evil was at work in these villages. Although such purges were greeted with natural scepticism and sometimes involved exclusion, LFA-led interventions were often deemed to keep the peace. To use Porter's terms, though these events carried the potential for exclusion, they were conceived as an important aspect of ‘social harmony’ (2016, p. i).

During our more recent fieldwork in Palabek, similar forms of exclusion were being perpetuated – not against strangers but against witchcraft. In the context of the anonymity of displacement camps it was anonymous healers, rather than villagers, who were associated with the threat of witchcraft. Such fears of witchdoctors made sense in the context of increasingly commercialised healing markets in Arua, Gulu and refugee settlements. The presence of these specialists was widely advertised on billboards, banners on homes and leaflets, offering cures for physical and mental afflictions, relationship problems or male impotency. Yet although the number of outsiders publicly engaging in healing has increased, Christian condemnation of witchdoctors has certainly – even ironically – raised their profile, creating a ‘moral panic’ (Allen, 2015) around the effects of their unverified services. Not infrequently, LFAs use the pulpit of church services to portray witchdoctors as the local personification of devil work. Their power is said to come from worshipping ‘idols’, rather than channelling godly forces (as faith actors do).

This condemnation has evolved into a sphere of public action against witchdoctors, now defined as a public health threat. In Arua, churches have formed new alliances with government actors and public health officials to cleanse the city of occult specialists. LFAs have been key instigators of ‘raids’ on witchdoctors’ homes, are deemed to have spiritual authority to destroy their ‘shrines’, and on occasion, have expelled them altogether (Storer, 2021). Importantly, these events create the public evidence of the evil that they describe, in the production of extensive paraphernalia – snakes, bones and wild animals—all emblems of evil. One event in 2016 that followed an outbreak of disease and fire in an urban slum, Oli, near Arua, reportedly resulted in the purge of more than 370 witchdoctors from their homes (Draku & Uganda Radio Network (URN), 2016). Similar events unfolded in Arua during 2018 (Amandu, 2018). A further significant turn is that, since this time, renowned Anglican leaders had recently begun working with the psychiatric unit at Arua Hospital, to spread word of the treatments among their congregation, as well as coming to the unit to pray with patients (Verginer & Juen, 2019). It seemed this formal inclusion within the health sector seemed to embolden social cleansing activities and bolster their personal legitimacy. These events in Arua offer a caution as to the exclusion that can result when faith actors’ spiritual claims become entangled with health advocacy.

As noted above, mental health professionals in the Palabek refugee settlement continuously struggled to establish themselves as legitimate actors in the local therapeutic marketplace. Formally, this difficulty was attributed by humanitarians to the ‘poor mental health literacy’ of the refugee population. Accordingly, psycho-education sessions took place at the main hospital in Palabek several times a week, to teach refugees about what the resident mental health nurse referred to as: ‘those [illnesses] that are disturbing your thoughts or your mind’. Yet, according to clinical staff these interventions seemed to do little to improve refugees’ reticence to consult the mental health clinic.

Although this reticence could stem in part from the limited relevance of Western concepts and treatments of mental illness in a setting like a refugee settlement, mental health practitioners in Palabek considered witchdoctors to be the cause of this lack of adherence to their programmes: ‘We lose so many patients to witchdoctors. When they find that the treatment has not been working, you find they disappear. That means they have “gone home” to treat the issue’, a psychiatric officer explained in frustration. Large portions of the sessions were thus dedicated to dissuading people from consulting witchdoctors when experiencing symptoms of mental illness. Bringing people to a witchdoctor, the same officer explained to refugees, was dangerous: ‘Those rituals can be harmful; some will cut people's skin, smear herbs on them using their bare hands and expose the person to infections or to pain that can expose the patient to new symptoms and create more complications.’ Several times, the nurse pleaded as well that: ‘If someone has those problems, bring them to the church, or tell them to come to the hospital, where there is a medication for that’. After listening to one such psycho-education session while waiting to see a doctor himself, a Christian pastor enthusiastically commented that he would immediately tell his congregants about the mental health clinic: ‘This way,’ he added, ‘they may go to the hospital when they are mentally ill, instead of to witchdoctors’. The management of mental health was thus brought into a complex marketplace, where LFAs used their legitimacy to disavow other healing specialists; to embed MPHSS, LFAs directly sought to cleanse communities of witchdoctors.

In Palabek, these community outreaches were as much an educational tool as they were an effort by humanitarian mental health workers to construct themselves as legitimate and trustworthy therapeutic actors in the eyes of the refugee population. To do this, psychiatric staff in Palabek chose to rely on the establishment of an explicit alliance between LFAs and humanitarian ones, in strong opposition to witchdoctors and other ‘traditional’ actors. In these speeches, mental health professionals therefore equated churches and hospitals with moral forces for good, and witchdoctors with forces for evil. The concern advanced by this research, however, is that historical evidence indicates that in contexts of affliction and death, castigations of witchdoctors have often turned into violent purges (Allen, 1991; Harrell-Bond, 1986). Our evidence suggest that these risks are magnified by novel and uncritical reliance by the humanitarian system on LFAs to deliver mental health.

## Discussion

When the humanitarian apparatus chooses to rely on faith-based actors, it chooses to do so for reasons related to facilitating its operations on the ground. The logic of convenience behind this choice is easy to grasp: seeking the support of individuals and institutions that retain legitimacy among the local population is in many ways a sensible antidote for the diffidence that humanitarians may experience, either towards themselves as external actors or towards the programmes they implement. Certainly, this choice is well-intentioned, and emerges from an awareness of longstanding and ongoing debates regarding the problematics of imposing externally defined priorities without local buy-in.

Yet our evidence shows that humanitarianism's engagement with LFAs frequently overlooks essential aspects of their social role in the communities where they exist and operate. Indeed, we have found it is precisely these historical engagements, such as those in attending to mental disquiet that have rendered faith actors able to act as intermediaries or ‘brokers’ between humanitarians and local populations, that are written out of policy calculations. Indeed, we contend that the prior social embeddedness of longstanding therapeutic engagement can in fact serve as significant barriers to instilling humanitarian visions of rights-based protections.

Based on our extended engagement in fields of healing in Arua and Palabek, we have shown how the management of mental affliction results from long processes, whereby Christianity was legitimised through proving the impotence of ancestors and diviners. It is significant, moreover, that these struggles have been emboldened because pastors and priests have long served as local anchors for international partnerships – secular and spiritual – with European and American churches, as well as with donor agencies. But on the ground, legitimacy has become entangled with entrenching – and reshaping – particular vernaculars of affliction, both mental and physical. Although these struggles may seem entirely remote to mental health professionals in Euro-American contexts, they have been replicated over many African (and other) contexts.

In contemporary times, we have shown how struggles over legitimacy include competition with biomedicine, and we have suggested that exclusionary practices connected to faith healing tend to be legitimated when external actors formally include faith actors in the delivery of health (as biomedically defined). Struggles in therapeutic marketplaces are always evolving – the arrival of, for instance Pentecostal and transnational fundamentalist Christian movements – which inevitably shift the contours of competition and local debate. We have located our findings within the wealth of anthropological literature, which has urged that Christianity specifically and faith generally be considered not as an abstract institution, but as a set of ideas and practices that are constantly enculturated into the moral, social and spiritual world of believers (Allen, 1991; Behrend, 2011; Wild-Wood, 2010). We thus urge a reconsideration of LFAs’ longstanding roles as both mediators and shapers of hybrid socio-spiritual worldviews, and as interested parties in healing marketplaces. This reconsideration has particular implications for their reiteration of normative patriarchal views entrenched through healing practices. As Tyszler notes with reference to the intersection of religious and humanitarian intervention in Morocco, religious actors often operate in ways aimed at ‘preserving exemplary moral standards of femininity’ (2019, p. 60). Here, too, their entanglement with humanitarian actors reinforces oppressive patriarchal structures already in place.

As recent studies have shown, the cleansing of post-conflict trauma and other forms of psychological suffering remains an arena within which social and resource struggles play out. Thus, although much research on northern Uganda reveals contests between epistemological stances, the clustering of studies around particular concepts, actors and places leaves many other realities unanswered. In particular, studies that focus on patient pathways, a single healer or church congregation, tend to remove the social context from interventions. Yet we argue that the social context within which therapies are delivered is essential to understanding individual recovery, as well as the inadvertent effects of novel alliances in delivering therapies.

Significantly, it is striking how the positive visions of faith-based healing (e.g., Ager et al., 2018; Harsch et al., 2021; Williams, 2021) stand in tension with ethnographic work that has highlighted the challenges of rebuilding communities after crisis. Prospects of rebuilding lives are deeply gendered – although women may be empowered in church services, once those services have ended – they may well face difficulties accessing recognition within normative clan-based structures. As Porter's (2016) study of Acholi women's attempts (or not) to seek redress for rape powerfully elucidates, visions of social harmony are both inclusionary and exclusionary. Scholars have kept largely silent on the management of witchcraft, yet it is the very same therapists governing social evictions who manage supposedly restorative spiritual cleansing. Critically, as others have argued (Allen, 1991; O’Byrne, 2017), these processes represent another face of reconstruction. Our evidence, in conversation with the rich anthropological literature that has emphasised the frictions between different actors with competing visions of social repair (Macdonald, 2017; Storer et al., 2017; Victor & Porter, 2017), suggests that these collective social processes equally link to mental health agendas in surprising ways.

Although we acknowledge the fundamental role of faith and religious communities in contributing to psychological wellbeing, we argue that the aspects of exclusion and reproduction of structural oppression that are inherent to the work of LFAs warrant concern. As the difficulties of localising external priorities for aid become apparent, scholars have often urged that the pre-existing attachments of local actors who serve as humanitarian partners be considered (Abramowitz & Kleinman, 2008). It seems surprising that the enthusiasm for faith actors persists even as scholars and activists campaign for a recognition of the exclusion practised within customary authorities (Quinn, 2022), as well as the violent roots of Christian imposition from the colonial period (Benthall, 2017). We suggest that it is only by engaging in this complexity that realistic dialogue can begin between actors to promote forms of mental health support that respect individual rights and dignity.

## Concluding remarks

Contemporary humanitarians advocate for a global mental health movement premised on human rights, individuated treatment and a recognition of the structural determinants of mental distress. Pursuing this laudable aim, it is assumed that local actors are conversant and invested in Western notions of mental health. Yet by neglecting the complex and fraught therapeutic landscape of northern Uganda, flows of humanitarian resources directed at LFAs may risk overlooking the intricate histories, relationships and agendas of these actors. Considering these factors and complexities, we caution against the recent enthusiasm that sees enormous potential in training and relying on LFAs to deliver mental health assistance.

In societies where identity is relational, and managing affliction has long involved relating symptoms to the activities of others, LFAs’ interventions are likely to be pulled into deeply embedded exercises to redefine social boundaries. Indeed, it is this deep historical involvement – borne through local populations’ navigation of colonial violence and post-conflict upheavals – that has rendered many LFAs legitimate arbiters to determine the cause of affliction. We suggest these activities cannot simply be ignored in favour of developing toolkits to instrumentalise imported concepts of mental health, but rather should constitute an important starting point to consider localising aid.

When social dynamics and longstanding histories of managing affliction are side-lined in humanitarian work, caution is warranted. Such interventions may exacerbate struggles for legitimacy in therapeutic marketplaces, where a variety of Anglican, Catholic, Pentecostal and other actors compete both among themselves and with traditional healers. At the centre of such struggles lies the management of mental disquiet, and, if these risks go unheeded, the potential for profound exclusion for sufferers.

## Notes

1. Although these humanitarian policy shifts are presented as widely innovative, the idea of mobilising faith actors in the provision of health care is not entirely novel. Winiger and Peng-Keller (2021) point out that in many ways, what we see today is akin to the World Health Organization (WHO)'s efforts to establish community-based models of health care drawing on traditional healers and midwives dating back to the 1960s and 1970s (see, e.g., Mahler 1977), and to subsequent several attempts to rely on faith actors for the provision of health care.
